# Modelling Tools to Characterize Acetaminophen Pharmacokinetics in the Pregnant Population

**DOI:** 10.3390/pharmaceutics13081302

**Published:** 2021-08-20

**Authors:** Sofie A. M. Brookhuis, Karel Allegaert, Lidwien M. Hanff, Marjolijn N. Lub-de Hooge, André Dallmann, Paola Mian

**Affiliations:** 1 Princess Maxima Centre for Pediatric Oncology, 3584 CS Utrecht, The Netherlands; l.m.hanff@prinsesmaximacentrum.nl (L.M.H.); p.mian@prinsesmaximacentrum.nl (P.M.); 2 Department of Development and Regeneration, KU Leuven, 3000 Leuven, Belgium; karel.allegaert@uzleuven.be; 3 Department of Clinical Pharmacology and Pharmacotherapy, KU Leuven, 3000 Leuven, Belgium; 4 Erasmus Medical Center (MC), Department of Hospital Pharmacy, 3015 GD Rotterdam, The Netherlands; 5 Department of Medical Oncology, University Medical Center Groningen, University of Groningen, 9713 GZ Groningen, The Netherlands; m.n.de.hooge@umcg.nl; 6 Pharmacometrics/Modeling and Simulation, Research and Development, Pharmaceuticals, Bayer AG, 51373 Leverkusen, Germany; andre.dallmann@bayer.com

**Keywords:** acetaminophen, pregnancy, pharmacokinetics

## Abstract

This review describes acetaminophen pharmacokinetics (PK) throughout pregnancy, as analyzed by three methods (non-compartmental analyses (NCA), population PK, and physiologically based PK (PBPK) modelling). Eighteen studies using NCA were reported in the scientific literature. These studies reported an increase in the volume of distribution (3.5–60.7%) and an increase in the clearance (36.8–84.4%) of acetaminophen in pregnant women compared to non-pregnant women. Only two studies using population PK modelling as a technique were available in the literature. The largest difference in acetaminophen clearance (203%) was observed in women at delivery compared to non-pregnant women. One study using the PBPK technique was found in the literature. This study focused on the formation of metabolites, and the toxic metabolite N-acetyl-p-benzoquinone imine was the highest in the first trimester, followed by the second and third trimester, compared with non-pregnant women. In conclusion, this review gave an overview on acetaminophen PK changes in pregnancy. Also, knowledge gaps, such as fetal and placenta PK parameters, have been identified, which should be explored further before dosing adjustments can be suggested on an evidence-based basis.

## 1. Introduction

During pregnancy, many anatomical and physiological changes are observed, such as increased total body water and fat, increased cardiac output, and increased renal blood flow and function [1]. These changes can significantly affect the pharmacokinetics (PK) of many drugs. Due to legal and practical considerations, pregnant women are often not included in clinical trials [2]. Therefore, little information is available regarding the influence of PK changes on dose requirements during pregnancy, for the majority of drugs (e.g., antibiotics, epileptics) [1]. However, knowledge about PK changes is essential to optimize pharmacotherapy in pregnant women [2].

**Figure 1:**
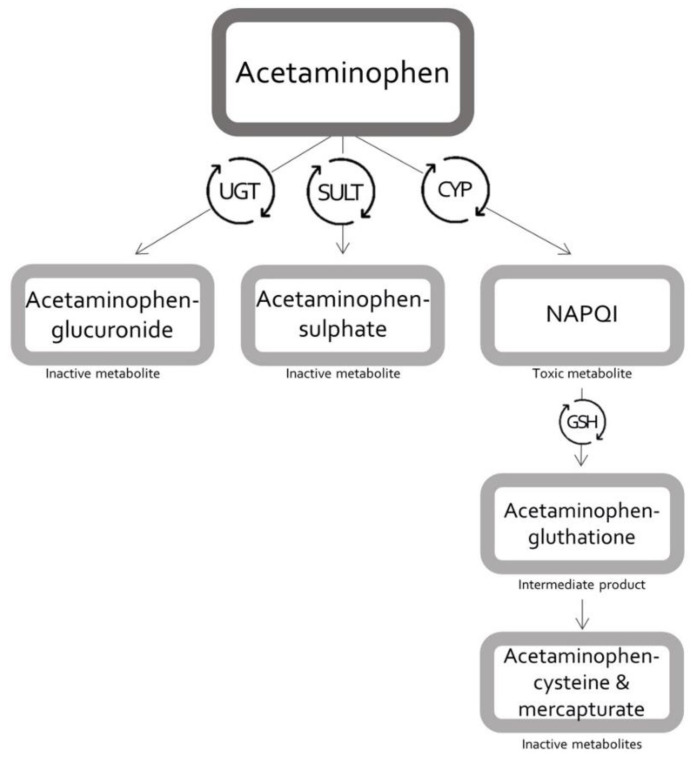
Schematic flow of acetaminophen metabolism. CYP = cytochrome P450; GSH = glutathione; NAPQI = *N*-acetyl-*p*-benzoquinone imine; SULT = sulfotransferase; UGT = uridine 5′-diphospho-glucuronosyl-transferase.

One of the most commonly used drugs during pregnancy is acetaminophen (paracetamol, APAP) and 40.5% of pregnant women use acetaminophen at least once [3,4]. During pregnancy, oral acetaminophen is used to treat headaches, migraines, back pain, or fever [4]. Intravenous (IV) acetaminophen is used, besides treating fever and pain, as analgesic during and following cesarean delivery, as part of multimodal analgesia [5]. In non-pregnant healthy adults receiving acetaminophen within the therapeutic dose range, acetaminophen is mainly metabolized through different uridine 5′-diphospho-glucuronosyltransferase (UGT) isoforms, mainly UGT1A1, to acetaminophen-glucuronide (55%), and through sulfotransferases (SULT), mainly SULT1A1, to acetaminophen-sulphate (30%) (Figure 1). Additionally, a minor fraction (5–10%) of acetaminophen is metabolized via several cytochrome P450 (CYP) enzymes, primarily by CYP2E1 and, to a lesser extent, by CYP2D6, yielding the toxic metabolite *N*-acetyl-*p*-benzoquinone imine (NAPQI). Also, a small fraction is excreted unchanged in urine (5%) [6,7,8]. Under normal conditions, NAPQI immediately reacts with glutathione, to form a glutathione adduct, and is thereby detoxified. This acetaminophen–glutathione conjugation is then metabolized to acetaminophen–cysteine and acetaminophen–mercapturate [9]. However, when glutathione becomes depleted (e.g., after an acetaminophen overdose or chronic alcohol abuse), NAPQI can form toxic protein adducts, which ultimately results in hepatocellular necrosis [7,10,11].

**Figure 2:**
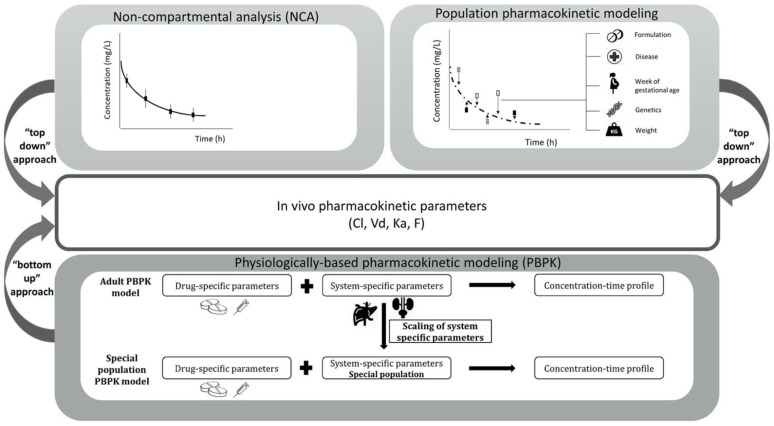
Pharmacometric tools used to support pharmacokinetic analysis in pregnant women, divided in the following two modelling approaches: “top-down” and “bottom-up” approach. “Top-down” approach is often referred to as non-compartmental analysis and population pharmacokinetic modelling, while “bottom-up” approach is physiologically based pharmacokinetic modelling. CL = clearance, Vd = volume of distribution, Ka = absorption rate constant, F = bioavailability.

The PK of acetaminophen in pregnant women has been studied across different trimesters of pregnancy and at delivery, using different PK analysis techniques [1,6,12,13,14,15,16]. Dosing in pregnancy is, among other things, dependent on the pregnant patient’s PK, which is typically quantified by determining different PK metrics. Therefore, these values should be reported for the studied population, together with the methodology used. PK values should be both accurate and generalizable to all individuals of a previously studied population [17]. The pharmacometric tools supporting PK analysis, which are often applied to special populations, can be roughly divided into two approaches. One is the data-driven method, this approach is based on observed drug concentrations in, e.g., blood samples originating from clinical studies or routine care. It reflects the “top-down” approach, as it allows for the calculation of the area under the concentration–time curve (AUC), which can be used to estimate PK parameters such as clearance (CL) (Figure 2). The “top-down” approach includes non-compartmental analyses (NCA), as well as the population PK modelling approach [18]. To date, most PK studies that are performed in pregnant women make use of intensive sampling and are analyzed using the NCA approach [1]. The changes in PK parameters during pregnancy are usually studied by expressing individual PK parameters per kilogram bodyweight, stratified by the trimesters of pregnancy [17]. The population PK modelling approach describes the PK changes of acetaminophen within pregnancy, at a population level. This modelling technique derives a concentration–time curve by pooling the blood samples from all the patients [19]. The other approach is known as the “bottom-up” approach, as its parameters are not informed by data, but are based on in vitro or in vivo experiments that measure these parameters. This “bottom-up” approach includes physiologically based pharmacokinetic (PBPK) modelling. PBPK modelling is a compartmental modelling approach, based on the physiological properties of the organism and the physicochemical properties of a drug. This modelling technique describes the interaction between a drug and the physiological system (organs/tissues) of the studied population, in a mechanistic manner, based on multiple ordinary differential equations [17,20]. However, PBPK modeling can also follow a “middle-out” approach when some model parameters are fitted to clinical PK data, while others are informed on the basis of in vitro or animal experiments [21]. The majority of PBPK models that are developed for pharmaceutical drugs follow this “middle-out” approach.

This review will describe acetaminophen PK throughout pregnancy, analyzed by three different PK modelling techniques, namely, NCA, population PK modelling, and PBPK modelling.

## 2. Materials and Methods

A PubMed search was conducted on 15 November 2020, to retrieve studies on acetaminophen PK throughout different trimesters of pregnancy or in women at delivery. No language restrictions were set. Keywords used were paracetamol/acetaminophen/propacetamol, pharmacokinetics, pregnancy/pregnant/gestation/perinatal/labor/labour/delivery. For inclusion, acetaminophen administration, by either IV or enteral (oral, rectal) routes, were considered. Only studies on acetaminophen in therapeutic dosages (0.65–2 g) were included. Studies comparing the PK of acetaminophen during pregnancy or at delivery with non-pregnant women or postpartum women were included, to determine the potential influence of pregnancy and/or delivery. No full systematic search on data in non-pregnant women or postpartum women using acetaminophen was performed, because this population acts as the comparison group. Studies eligible for this review were randomized controlled trials, observational studies, and case reports. In addition, references of included studies were checked for relevant articles. The obtained publications were categorized, as they were analyzed, according to the applied analysis technique, namely, (1) NCA, (2) population PK modelling, or (3) PBPK modelling. For each of these techniques, the PK changes of acetaminophen in pregnant women, estimated by the technique, are presented.

## 3. Results

### 3.1. Pharmacokinetic Estimates Obtained with Non-Compartmental Analyses

#### 3.1.1. Characteristics of the PK Studies

There were 18 studies on acetaminophen PK, using NCA methods, included [12,13,14,22,23,24,25,26,27,28,29,30,31,32,33,34,35,36]. Table 1 provides the characteristics of the included PK studies. Among these studies, 7 studies included pregnant women in their first trimester, 5 in their second, 9 in their third trimester, 4 studies included women at delivery, and 7 included postpartum women. The number of subjects included in these studies ranged from 2 to 96. When all the studies were combined, the numbers of subjects during their first, second, third trimester, at delivery, and postpartum were 88, 38, 102, 82, and 99, respectively. The mean or median age and weight varied from 21 to 32 years and 55.4 to 123 kg, respectively. The PK parameters derived from literature are provided in Table 2, Table 3 and Table 4 for the individual studies. Using the absorption, distribution, metabolism and elimination (ADME) sequence, the results of these studies are summarized below.

#### 3.1.2. Influence of Pregnancy on Acetaminophen Absorption

Of all 18 studies that applied traditional methods, 14 reported absorption-related parameters [12,13,14,22,23,25,26,27,29,32,33,34,35,36]. None of these studies compared, longitudinally, IV with the oral administration of acetaminophen, or reported on the oral bioavailability of acetaminophen in a paired analysis. The maximum concentration (C_max_) and time at which the C_max_ is achieved (t_max_), are often considered to be absorption-related PK parameters. However, these are not solely dependent on the absorption phase, but also on, e.g., first-pass metabolism and distribution. To be consistent with the literature, the information per individual study on t_max_ and C_max_ is reported in Table 2, Table 3 and Table 4 (subheading absorption-related parameters). At a dose of 1500 mg, the absolute C_max_ decreased significantly, by 22.1% (*p* < 0.05), in the first trimester of pregnancy compared to non-pregnant women [29]. In the second trimester of pregnancy, the absolute C_max_ of acetaminophen significantly decreased, by 37.8% (*p* < 0.05), compared to non-pregnant women [34]. No significant change in C_max_ in the third trimester of pregnancy, compared to non-pregnant women, was reported. In contrast to the C_max_ values, t_max_ increased significantly in the first trimester of pregnancy, by 43.8% (*p* < 0.05) [29]. In the second trimester of pregnancy, t_max_ increased significantly, by 59.8% (*p* < 0.05), compared to non-pregnant women [34]. Again, no significant change for the t_max_ values in the third trimester of pregnancy was reported.

In conclusion, both C_max_ and t_max_ values vary widely between all the different subjects studied, even when corrected to dose, as shown in Table 1.

#### 3.1.3. Influence of Pregnancy on Acetaminophen Distribution

**Figure 3:**
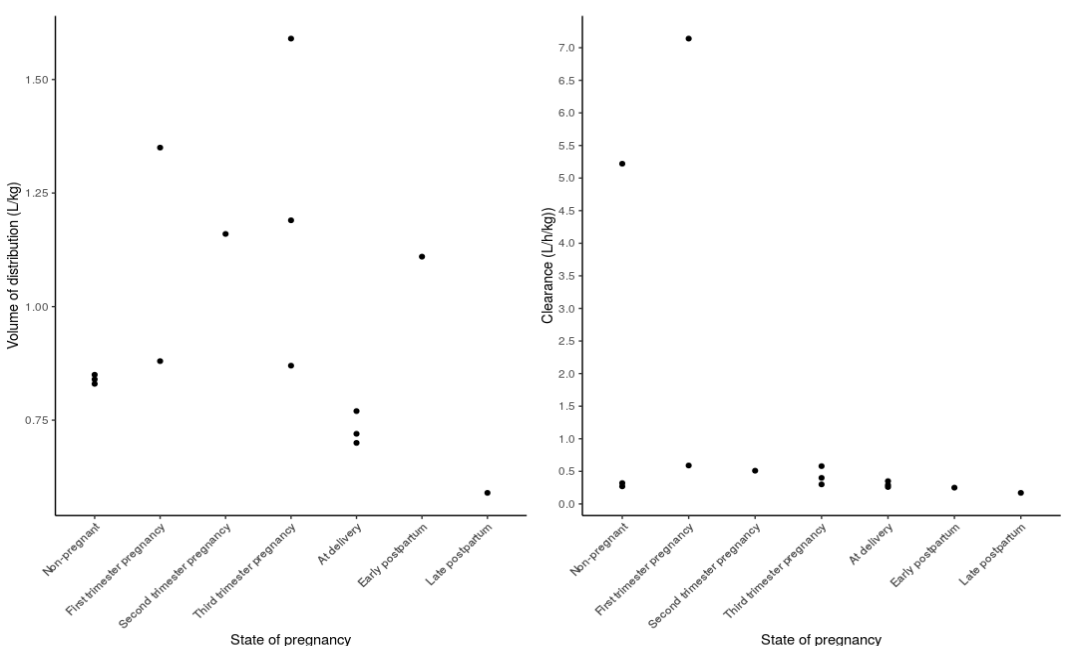
Scatter plots of the measured volume of distribution (expressed in L/kg) and clearance (expressed in L/h/kg) obtained from non-compartmental analysis techniques in non-pregnant women, first-, second- and third-trimester pregnant women, women at delivery, and early and late postpartum women.

Only five studies reported on the volume of distribution (Vd) of acetaminophen [12,22,27,30,31]. Another two studies did not report Vd [28,32], but the Vd was calculated based on the reported CL/F and half-life (t ^1^/_2_). The information per individual study on Vd is reported in Table 2, Table 3 and Table 4 and shown in Figure 3. Of these seven studies that report Vd, three [12,22,28] reported an increased Vd when comparing non-pregnant women with pregnancy. However, this pregnancy-related increase in Vd was not significant. One of these studies, by Beaulac-Baillargeon et al. [12], compared the Vd longitudinally in the same woman, prior to pregnancy, in her first (60.7% increase), second (38.1% increase), and third (41.7% increase) trimester. Another study by Beaulac-Baillargeon et al. [22] compared non-pregnant women with pregnant women in the first trimester (3.5% increase, non-significant (ns)). The third study, by Miners et al. [28], compared non-pregnant women with third-trimester pregnant women (4.8% increase, ns).

The Vd at delivery was reported by three studies [27,30,31]. When these Vd values are compared with the other studies investigating Vd throughout pregnancy or non-pregnant women, the Vd was found to be decreased at delivery. This finding is supported by Rayburn et al. [32], who reported a decrease, by 30.2%, in Vd when comparing early postpartum women with third-trimester pregnancy. The increased Vd in pregnancy can be physiologically explained by pregnancy-related increased plasma volume, increased total body water, and increased body fat during pregnancy [37]. The relative hydrophilic character of acetaminophen, together with the increase in body water in pregnancy, could cause the Vd to increase with pregnancy. However, it has to be noted that a large variability in values between studies has been reported. For example, when comparing the three studies reporting on third-trimester pregnant women [12,28,32], the values of Vd varied between 0.87 and 1.59 L/kg.

In conclusion, it is reasonable to state that the Vd of acetaminophen increases as pregnancy progresses. After delivery and during the postpartum period, the Vd values decrease again. Both statistical significances and clinical relevance are difficult to interpret, due to the studies’ limitations and due to the limited comparison of all the different stages of pregnancy with one another. All the studies report comparisons between the different trimesters. However, changes in the Vd values of acetaminophen in pregnant women are important to distinguish, because, theoretically, the Vd determines, amongst others, the loading dose. Although, it has to be noted that adjusting a loading dose, only based on changes in Vd, will be difficult in clinical practice, since it only takes changes in PK into account, but does not consider pharmacodynamics (PD).

#### 3.1.4. Influence of Pregnancy on Acetaminophen Metabolism and Elimination

There were seven studies that reported the CL/F or CL of acetaminophen [12,22,27,28,30,31,32]. The information of each study on CL/F or CL is reported in Table 2, Table 3 and Table 4. When comparing non-pregnant women with all the trimesters of pregnancy, an overall increase in acetaminophen CL/F was observed, which further increases with increasing trimester. In the first trimester, CL/F increases significantly, by 36.8% (*p* = 0.03), compared to non-pregnant women [22]. In the second trimester, an increase in acetaminophen CL/F of 59.4% was observed in one woman who was also studied prior to pregnancy [12]. The CL/F of acetaminophen significantly increased, by 58.3% (*p* < 0.002), in the third trimester compared to non-pregnant women [28]. The increased CL/F of acetaminophen in pregnant women can physiologically be explained by the increased enzyme activity of, e.g., UGTs and CYPs, which are driven by hormonal changes, and by a higher glomerular filtration rate during pregnancy [1,38].

Three studies reported the CL/F and CL of acetaminophen at delivery [27,30,31], and reported lower CL/F and CL values (0.26–0.35 L/h/kg) compared with the other studies investigating the CL/F of acetaminophen throughout pregnancy or non-pregnant women [12,22,27,28,30,31,32]. This finding is supported by Rayburn et al. [32], who reported a decrease (16.7%) in acetaminophen CL/F after delivery, comparing women in the third trimester with early postpartum women. Only one study compared late postpartum women with women at delivery and reported a significant decrease in acetaminophen CL/F (41.4%, *p* = 0.0078) in the late postpartum women [31]. This change in the CL/F of acetaminophen might be related to changes in intrinsic enzyme activity driven by hormonal changes [1,38]. None of the included studies reported longitudinal changes in acetaminophen CL/F in both postpartum and non-pregnant women.

Only three studies [24,28,32] focused on the contribution of the different metabolic routes on the elimination of acetaminophen within pregnancy. One of these studies compared third-trimester pregnancy with non-pregnant women [28], while the others compared third-trimester pregnancy with postpartum women [24,32]. All of these studies reported changes in the contribution of the different metabolic routes within the third trimester. However, these studies show conflicting results on the percentages of the changes in acetaminophen metabolites from the third trimester to 2–6 weeks postpartum, and from the third trimester to non-pregnant women. Miners et al. [28] reported an CL/F increase of 58% in third-trimester pregnant women compared to non-pregnant women, due to increased glucuronidation, by 75%, and an 88% increase in metabolism through oxidative pathways. No significant differences in the formation CL/F of unchanged acetaminophen and acetaminophen-sulphate were observed. On the other hand, Galinski et al. [24] reported a decrease in the urine excretion of unchanged acetaminophen (2% vs. 5%) and acetaminophen-sulphate (33% vs. 44%), and also an increase in acetaminophen-glucuronide (65% vs. 51%) in third-trimester women compared to early postpartum women. A limitation of the study by Galinksi et al. [24] is the assumption that acetaminophen is converted to either acetaminophen-sulphate or acetaminophen-glucuronide, or is excreted as an unchanged drug, not considering metabolism through oxidative pathways. Rayburn et al. [32] found no significant changes in the urine excretion of any of the acetaminophen metabolites. It should be taken into account that the results will depend, amongst others, on the study group size, the duration of sample collection, and the amount of repeated measurements, which is different in all three studies (Table 1).

As a secondary PK parameter, t^1^/_2_ is related to Vd and CL. There were nine studies that reported on the t^1^/_2_ of acetaminophen in pregnancy [12,22,27,28,30,31,32,35,36]. The information on t^1^/_2_ values is reported in Table 2, Table 3 and Table 4. An overall decrease in t^1^/_2_ has been observed in pregnancy, when comparing non-pregnant women to different stages of pregnancy. In the first trimester, a significant decrease of 19.8% (*p* < 0.005) has been observed, compared to non-pregnant women [22]. One woman, in the second trimester pregnancy, showed a decrease of 13.6% compared to the same woman before pregnancy [12]. In third-trimester pregnancy, t^1^/_2_ has significantly decreased by 28.0% (*p* < 0.002) compared to non-pregnant women [28]. This decrease in t^1^/_2_ in pregnancy is in line with the reported increase in CL/F.

The wide ranges in reported PK values, relating to absorption, distribution, and elimination, may be explained by the wide variety of patient characteristics, study group size, sampling, and dosing schemes, between the analyzed studies, type of delivery (natural vs. cesarean section), and potential co-medication(s) (e.g., affecting GI function) (Table 1). Additionally, the range in PK values may be explained by the data analysis method used (NCA). Most of the analyzed studies are based on relatively small numbers of subjects within each group (Table 1), limiting the precision of each PK value and its true population variability.

In conclusion, acetaminophen CL/F increases during pregnancy, with advancing trimester. Based on limited data, CL/F seems to decrease again after delivery and in early postpartum women. Changes in the formation of acetaminophen metabolites are only investigated in the third trimester. Concluding from these results, the contribution of the different metabolic routes to total clearance is different in pregnancy compared to non-pregnant women. Also, the results suggest that early postpartum women do not have a similar CL/F of acetaminophen vs. women in the late postpartum phase. However, it has to be noted that these two stages of pregnancy have not been studied in direct relation to one another, but each postpartum period has been reported in a different study.

### 3.2. Pharmacokinetic Estimates Obtained with Population Pharmacokinetic Modelling

Characteristics of the two population PK models that describe acetaminophen PK, discussed in this review, are summarized in Table 5. The first model, by Kulo et al. [15], comprised acetaminophen as well as unchanged acetaminophen, acetaminophen-glucuronide, acetaminophen-sulphate, and oxidative metabolites in urine. This model is based on data from 39 pregnant women, of which the data were modelled with the data of eight of these pregnant women 10–15 weeks postpartum, to study the difference in the PK of acetaminophen between these two groups. The patient characteristics are shown in Table 5.

This first model showed that the most significant covariate was delivery vs. postpartum, on the formation CL of acetaminophen-glucuronide (11.6 vs. 4.76 L/h), oxidative metabolites (4.95 vs. 2.77 L/h), unchanged acetaminophen (1.15 vs. 0.75 L/h), and on the central Vd of acetaminophen. No differences in the formation CL of acetaminophen-sulphate were observed. None of the other covariates (e.g., BW, weeks of GA) had an influence. It has to be noted that this study also had limitations. For instance, the Vd of acetaminophen-glucuronide and acetaminophen-sulphate were fixed to 18% of the central Vd of acetaminophen in plasma, to be able to estimate the fractions of acetaminophen to its metabolites [39]. This assumption was made because no urine data of acetaminophen-glucuronide and acetaminophen-sulphate were available. Therefore, the estimated percentages of these formations are specific to the stated assumption. Different techniques were used for internal validation, as shown in Table 5. Although, it has to be noted that no external validation of the model was performed.

The second model, by Allegaert et al. [16], was based on data from 69 young women (Table 5). Similarly to Kulo et al. [15], this model found that delivery was the most significant covariate for formation CL to acetaminophen-glucuronide (factor 2.03 higher CL at delivery vs. postpartum). The women at early postpartum had decreased formation CL of acetaminophen-glucuronide (factor 0.55) compared to non-pregnant women. Formation CL to acetaminophen-sulphate, at delivery, was not a significant covariate. The formation CL of acetaminophen to acetaminophen-sulphate was higher in pregnant women who delivered preterm (GA < 37 weeks, factor 1.34) compared to women with term delivery or non-pregnant women. The CL of unchanged acetaminophen was dependent on the urine flow rate, although this was not a significant covariate. Late postpartum (1 year later) did not prove to be a significant covariate, suggesting that the PK changes during pregnancy normalize after no more than 1 year of being postpartum and equalize to the pre-pregnant level. Similar to the model by Kulo et al. [15], a limitation of this model is that the Vd of acetaminophen-glucuronide and acetaminophen-sulphate was fixed to 18% of the central Vd of acetaminophen [39]. Also, formation CL, through oxidative pathways, was not implemented in this model, therefore the toxic oxidative metabolite NAPQI was not included. Both the models show variability in the diagnostic plots, which could not be explained by the studied covariates. This suggests that there are other, not yet studied, factors that further affect the PK of acetaminophen in pregnancy. Model validation was performed in the same way as the model by Kulo et al. [15]. Another remark is that both models use the same data, but both studies compare different subjects. The model by Kulo et al. [15] compares women at delivery with postpartum women, while the model by Allegaert et al. [16] is expanded, with comparison of healthy female volunteers, either on or not on oral contraceptives.

In conclusion, delivery was found to be the most significant covariate for the formation CL of acetaminophen-glucuronide and the central Vd of acetaminophen. Formation CL to acetaminophen-glucuronide increased when being at delivery vs. postpartum.

### 3.3. Pharmacokinetic Estimates Obtained with Physiologically Based Pharmacokinetic Modelling

**Figure 4:**
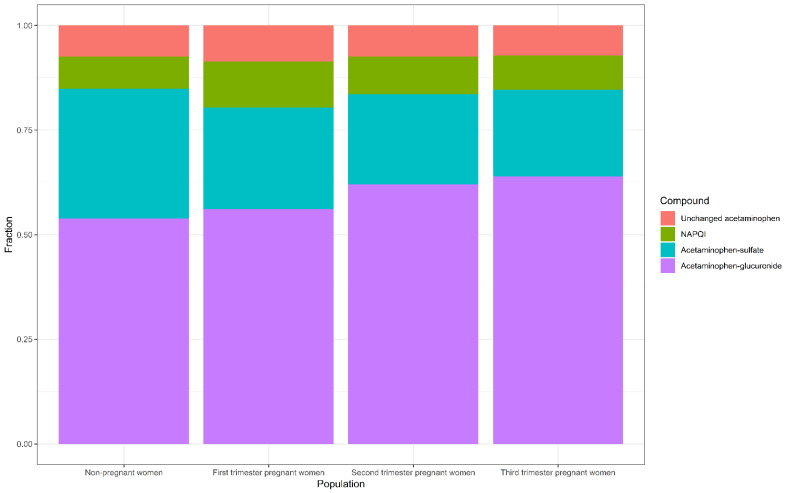
Barplot of the predicted molar dose fractions of unchanged acetaminophen excreted in urine and acetaminophen metabolites for non-pregnant women, and first-, second- and third- trimester pregnant women, obtained from PBPK analysis. Median fractions are shown. NAPQI = N-acetyl-p-benzoquinone imine.

One publication describing the PK of acetaminophen (and its metabolites) in the pregnant population, using PBPK modelling, was retrieved [6]. This model was compared to in vivo data of acetaminophen in pregnant women in the first and third trimester, as well as to women at delivery. It was mainly built with the purpose to describe changes in the formation CL of acetaminophen metabolites throughout pregnancy. Changes in UGT1A1, SULT1A1 and CYP2E1 enzyme activity throughout pregnancy, built in this model, were obtained from the literature [15,28,40,41,42,43]. The implemented pregnancy-induced changes in UGT1A1 activity relied on reported in vivo serum trough concentrations of bilirubin, an exclusive substrate of UGT1A1 [40]. The free-bilirubin trough concentration was, on average, 25%, 43%, and 48% lower in the first, second and third trimester, respectively, compared to that of non-pregnant women [41]. These decreases were assumed to relate directly to increases in UGT1A1-mediated bilirubin CL during pregnancy. SULT1A1 activity changes were obtained from reported activity changes in human endometrial tissue in early pregnancy, compared to the tissue of non-pregnant women. SULT1A1 activity did not differ between these subjects [42,43]. Parametrization of CYP2E1 activity was achieved by studying changes in the CL of acetaminophen in peripartum women, shortly after delivery, compared to 12-weeks postpartum women. In peripartum woman, the oxidative CL of acetaminophen was 1.8-fold higher than shortly after delivery [15,28]. This 80% increase that was observed in third-trimester pregnant women was extrapolated throughout the whole duration of the pregnancy. This was conducted because of the lack of data in first- and second-trimester pregnant women, assuming a ‘worst-case scenario’ by deliberately implementing in the model a bias towards higher CYP2E1 activity at earlier stages of pregnancy. Using the PBPK model, predictions were performed to estimate the molar dose fraction of acetaminophen converted to its metabolites (Figure 4).

Formation CL to NAPQI was used as a measurement for potential hepatotoxicity, although it should be noted that toxicity also depends on additional factors, such as the hepatocellular glutathione amount in pregnant women. The estimated molar dose fraction of NAPQI was the highest in the first trimester (median (IQR): 11 (9.1–13.4%), followed by the second (9.0 (7.5–11%)) and third (8.2 (6.8–10.1)) trimester compared with non-pregnant women (7.7 (6.4–9.4%)). The estimated molar dose fraction of acetaminophen-glucuronide was the highest in third-trimester pregnant women (63.9 (61.7–67.1%)), followed by the second (62.1 (59.3–65.1%)) and first trimester (56.2 (53.4–59.6%)), compared with non-pregnant women (53.8 (51.2–57.2%)). The estimated molar dose fraction of acetaminophen-sulphate decreased with the duration of pregnancy (24.2 (22.3–26.2%), 21.5 (19.9–23.1%) and 20.7 (19.1–22.4%) in the first, second and third trimester, respectively) compared with non-pregnant women (31.1 (29.0–33.3%)). The molar dose fractions of unchanged acetaminophen increased in first-trimester pregnant women (8.6 (0.9–13.5%)) compared to non-pregnant women (7.4 (0.1–13.5%)), after which it decreased in the second (7.4 (0.8–13.3%)) and third trimester (7.2 (0.3–12.3%)). For evaluation of this PBPK model, goodness-of-fit plots were used, amongst other diagnostics, to compare the predicted plasma concentration values to in vivo concentrations obtained from three different studies [16,22,44]. This showed a good agreement of the simulated plasma concentration–time profile of acetaminophen for both pregnant and non-pregnant women. Due to a lack of in vivo data on the oxidative metabolites, the predictions of NAPQI serum concentrations could not be evaluated.

In conclusion, the molar dose fraction of acetaminophen converted to NAPQI was highest in the first trimester, followed by the second trimester, third trimester, and non-pregnant women.

## 4. Discussion

In this manuscript, changes in the most relevant PK parameters of acetaminophen throughout pregnancy have been described. The advantages and disadvantages of three different PK techniques (NCA, population PK modelling, and PBPK modelling) have been compared and explored. Acetaminophen PK changes during pregnancy have been widely studied, with a relatively large amount of research applying NCA. The majority of NCA were performed near the end of (term) pregnancy, and the two included population PK models included women at delivery and in early postpartum. A probable cause of this large amount of data at the end of pregnancy is that these women are commonly in a hospital setting for delivery, which is associated with the standardized administration of acetaminophen. Only three of the NCA studies and the PBPK model covered all three trimesters of pregnancy.

Although the NCA approach is the most applied approach in this review, it also has limitations. For instance, this method uses stratification, meaning that it indicates only the differences between the different trimesters and not gestational age as a continuous variable. Also, this method requires a large number of samples per individual to cover the complete individual concentration–time profile, making this method not feasible for vulnerable patients, such as pregnant women. Another limitation is the need of a strict sample collection protocol (unbalanced data cannot be used) to determine an AUC per individual. Also, inter-individual variability (variability between subjects) and intra-individual variability (variability within one subject, measurement error, and model misspecification) cannot be distinguished from one another [17,45]. Therefore, other methodologies may be preferable and have been used in this population more recently.

Population PK modelling, on the other hand, allows for analysis with relatively dense, sparse and/or unbalanced data, compared to the NCA, making the population PK modelling approach more feasible to use for vulnerable patients, such as pregnant women. Also, this approach can be used for the meta-analysis of data from multiple studies with different designs [17,19]. The population PK approach can distinguish inter-individual variability from intra-individual variability. Also, this method allows for the identification of covariate relationships that describe continuous pregnancy progression, where NCA are typically stratified by trimester [17]. The disadvantages of this technique include that it still requires blood samples. Also, this type of modelling is a more advanced technique, which requires skilled professionals to perform it [17].

PBPK models, on the other hand, integrate many different tissues and organs as compartments, and kinetic data obtained from samples other than from blood, for instance cerebrospinal fluid, amniotic fluid, umbilical cord blood or urine, can be leveraged in these models. However, sampling is not a necessity, as this approach can also be used in a purely predictive manner, which is especially beneficial for a vulnerable population, where samples are difficult to obtain, such as pregnant women [17]. Another advantage of this modelling technique is that it describes PK mechanistically, making it possible to extrapolate data between populations, but also between drugs with similar PK mechanisms [6,17]. Also, these models are suitable to generate and test hypotheses on, e.g., physiological changes in the absence of clinical data. However, a limitation of this approach, compared to population PK modelling, is that it requires a wide variety of knowledge of physiological processes, which might be difficult to quantify. For example, there are still gaps in our knowledge on human physiology and enzymatic changes (e.g., expression and activity of UGT, SULT and CYP enzymes) during pregnancy, in both the mother and fetus [6,17]. Also, the evaluation of a PBPK model is based on a less statistical ground than, for example, a population PK model [17].

When looking at the changes in acetaminophen PK during pregnancy, the results of this review report an increase in acetaminophen CL/F in the first (36.8%), second (59.4%) and third trimester (58.3%) compared to non-pregnant women, as estimated by NCA. Acetaminophen CL/F is highest at delivery, after which it decreases during the postpartum period to values lower than those of pre-pregnant women or of women in the late postpartum period. This increase in acetaminophen CL/F seems to be mainly driven by an increased glucuronidation rate. When focusing on acetaminophen glucuronidation, Miners et al. [28] reported an increase of nearly 75% in the formation CL/F of acetaminophen-glucuronide in the third trimester of pregnancy, compared to non-pregnant women [28]. The results of the two included population PK models are in line with the results of the NCA.

Based on data on pregnancy-related alteration in free-bilirubin serum levels, the PBPK model confirms the increase in acetaminophen-glucuronide formation CL in pregnant women, with increasing trimester, compared to non-pregnant women. However, the limitations of this PBPK model are that the CL parameterization has been divided into three trimesters, but changes in clearance during pregnancy should not necessarily be discrete, but it should be a continuous progression. Second, data on CYP2E1 enzyme expression throughout human pregnancy are scarce, therefore changes in expression near term (80% increase) are used to describe changes throughout the whole pregnancy. According to this conservative approach, the high CYP2E1 activity, that is observed near term, translated into relatively high estimates of molar dose fractions of NAPQI at earlier stages of pregnancy. Different CYP2E1 activities within first and second trimester may be plausible. Therefore, lower CYP2E1 activities at earlier stages of pregnancy could result in a lower molar dose fraction that is oxidized to NAPQI. Third, while the changes in UGT1A1 activity, calculated from the in vivo serum trough concentrations of bilirubin, appeared to translate into adequate clearance estimates, this approach still has some limitations. For example, the main assumptions of this approach were that free-bilirubin trough concentrations correlate exclusively with changes in UGT1A1 activity. For example, it is unclear if bilirubin production rate and the related red blood cell turnover is changed in pregnancy. Another assumption is that UGT1A1 is the sole isoform involved in acetaminophen glucuronidation and that relative changes in bilirubin trough concentrations translate directly into changes in UGT1A1 activity. Also, this model only describes SULT1A1 expression, while other SULT isoforms might play a part in the CL of acetaminophen [6]. Once further data become available, they could be integrated in the PBPK model, to refine the previously reported PK predictions. When focusing on the increased overall CL/F of acetaminophen in pregnant women, this can physiologically be explained by the increased enzyme activity of, e.g., UGTs and CYPs, which are driven by hormonal changes, a disproportional increase in glucuronidation CL, and a proportional increase in the CL of unchanged acetaminophen and oxidative metabolites. [15,38]. For instance, Beleyn et al. [38] found a correlation between acetaminophen CL and estradiol, as well as progesterone, which qualitatively corroborates the hypothesis that the induction of these enzymes during pregnancy is driven by changes in hormone levels. Also, the use of oral contraceptives, obviously limited to non-pregnant women, affects the CL of acetaminophen [16].

After delivery, both hormone levels and body fat volume do not (immediately) return to the pre-pregnant level. Body fat volume, for instance, remains elevated, which might be explained by the increased energy demand during breast feeding [38,46]. These physiological changes in postpartum women might explain the reported differences in the CL/F of acetaminophen between early postpartum women and prepregnant women. No significant differences in acetaminophen CL/F were found in late postpartum women (>1 year) compared to pre- or non-pregnant women, suggesting that the PK changes during pregnancy normalize after no more than 1 year of being postpartum and equalize to those observed in prepregnant women.

Although this review reports a breadth of different data on acetaminophen PK parameters during pregnancy, it has to be noted that some data are still lacking or sparse, such as observations in first and second trimester of pregnancy, or information on the oxidative metabolites throughout the entire duration of pregnancy. Since all these concentration–time points or profiles from the different NCA, as well as population PK models, have been derived already, it might be possible to pool all this data to form one group of subjects, to perform a meta-analysis to identify exactly which data are lacking. For instance, all women who were included in the studies were healthy. It has to be noted that in the real-world, not all pregnant women are healthy, and some can suffer from various conditions, e.g., preeclampsia, deliver preterm, or experience twin or triplet pregnancy. It would be representative for the pregnant population to develop PBPK models that integrate disease-related effects on physiological parameters and subsequently collect clinical data.

Besides the obtained insights into the differences in the PK parameters of acetaminophen during pregnancy, information on acetaminophen PD in this population is necessary, to suggest dose adjustments. Since a strong correlation between acetaminophen concentration and analgesia is proven in healthy volunteers [47,48], a higher CL/F of acetaminophen in pregnant women assumes that these women experience a less analgesic effect than non-pregnant women. However, before recommending to increase the dose of acetaminophen in this population, to compensate for the higher CL/F, it has to be noted that the molar dose fractions of the toxic metabolite NAPQI might be increased in pregnant women [6,15]. It is likely that this toxicological aspect limits higher doses of acetaminophen in this population, on the basis of safety considerations. Because information on acetaminophen PD in pregnant women is still missing, it raises the question of whether pregnant women really do experience inadequate analgesia, or if this is purely an assumptive derivative of the changes observed in the PK parameters. Further research, with a focus on the PD mechanism of acetaminophen, is needed, to elucidate the potential changes therein during pregnancy and to achieve adequate analgesic effects. Until such data become available, it is reasonable to assume that the higher clearance will result in a faster decrease in analgesia (duration, level of analgesia), which cannot simply be compensated by higher dosing, as this will likely result in more toxicity. While this review focused only on changes in the PK parameters of acetaminophen in the maternal compartments, the fetal and placental PK of acetaminophen also has to be considered before dose adjustments can be recommended. According to the pregnancy risk categories that were used by the US Food and Drug Administration, prior to 2015, acetaminophen is classified as pregnancy category B for oral administration [49] and category C for IV administration [50], which means fetal risk cannot be ruled out. The use of acetaminophen in pregnancy has been associated with increased risks of attention-deficit activity disorder (ADHD), asthma in childhood, reduced fertility, and fetal ductus arteriosus constriction or closure [3,27,51]. However, the extent of fetal exposure to acetaminophen in maternal use from a standard dose, is still not fully understood [27,52]. Nitsche et al. [27] reported that fetal acetaminophen PK parallels the PK of acetaminophen within the maternal system. The values for t_max_ and t_1/2_ are similar in the fetus compared to those of the mother, and the fetal AUC is close to the maternal AUC (>90% similarity). This comparison suggests that the placental transfer of acetaminophen occurs primarily by passive diffusion and is not influenced by placental transporters [27]. Therefore, the authors suggested taking maternal plasma acetaminophen levels as a surrogate for fetal exposure. However, the different metabolites were not considered [3,27]. Furthermore, fetal APAP toxicity can also be explained by the direct impact on the placental functions. The placenta is an endocrine organ in itself, with relevant prostaglandin synthesis excreted to the fetal circulation, and this synthesis is likely affected by APAP exposure [53].

Mian et al. [54] developed a PBPK model to predict the placental transfer of acetaminophen, as well as the specific CL pathways in a term fetus. The expression and activity of UGT1A1 and CYP2E1 enzymes are significantly lower in the fetal liver (0.1–1% and 16.2% of the adult liver, respectively) [55,56]. SULT expression in the fetal liver, on the other hand, is reported as 6.5-fold higher than in the adult liver. Besides, Mian et al. [54] described that acetaminophen diffusion back to the mother can be considered the main pathway for acetaminophen elimination in the fetus. However, a limitation of this developed model is that these CL pathways of acetaminophen within the fetus are only described for a term fetus. For the earlier stages of pregnancy, these pathways can be different because of the development of the fetus and placenta [54]. This will affect the amount of acetaminophen that is transferred across the placenta and the subsequent fetal exposure. CYP2E1, which is responsible for forming the toxic metabolite NAPQI, was expressed in the placenta in 84% of the tested samples, suggesting that this could also affect acetaminophen PK in pregnancy. However, it has to be noted that the expression of CYP2E1 was significantly lower in the placenta as compared to the adult liver (factor 4380) [57]. To get a complete overview of acetaminophen PK parameters in pregnancy, the placental transfer of acetaminophen metabolites and placental metabolism have to be explored further. Ideally, the effects of maternal ethnicity, pregnancy-related and non-related co-morbidity, and concomitant medication on acetaminophen PK and PD also should be further considered.

## 5. Conclusions

NCA, population PK, and PBPK modelling have been applied to study acetaminophen PK in pregnant women. These techniques describe the increased CL/F of acetaminophen within the pregnant population compared to non-pregnant women. Also, the formation CL of the toxic metabolite NAPQI might increase in pregnancy compared to non-pregnant women. Ideally, the advantages of these different techniques should be combined, to advise dose regimens of acetaminophen in pregnant women in the future. Also, fetal PK and placenta PK parameters should be explored both in silico and in vivo, before dosing adjustments can be suggested on an informed basis. At least, this analysis illustrates the feasibility to use different modelling techniques to educate on changes in PK during pregnancy, for the most commonly used drug.

## Figures and Tables

**Table 1:** Overview of characteristics of included PK studies, obtained with NCA. Studies are presented in alphabetical order by author. Data shown as median [range] or mean (SD) otherwise if specified.

Reference	Patient Population Number	Weight (kg)	Age (Years)	Condition	Trimester	Form	Dose	Sampling (Sampling Period)	Sampling Duration	Analytical Method
**Beaulac-** **Baillargeon et al. (1993)** [12]	**NP = P1 = P2** **= P3:** 1	**NP**: 72 **P1**: 74.5 **P2**: 78.5 **P3**: 88.5	**NP**: 23 **P1 = P2 = P3**: 24	Non-smoker, no other medication, sometimes headaches, fasted for 3 h before APAP intake	**NP**: - **P1**: 12 weeks GA **P2**: 20 weeks GA **P3**: 30 weeks GA	Oral tablet	0.65 g (with 200 mL water)	*Blood:* Sampling on fixed times TOT: 52 Per patient:13 (0–4 h)	4 h	HPLC
**Beaulac-Baillargeon et al. (1994)** [22]	**NP**: 10 **P1**: 8	**NP***:* 60 **P1***:* 62.5	**TOT**: 28.3 (2.1) ^a^	**All**: healthy, no interfering medication **NP**: fasted for 3 h **P1**: undergoing abortion or had headache. Fasted before abortion.	**NP***:* - **P1***:* 11.1 (0.5) ^a^ weeks GA	Oral tablet	0.65 g (with 200 mL water)	*Blood:* Sampling on fixed times TOT: 216 Per patient: 12 (0–4 h)	4 h	HPLC
**Clark and Seager (1983)** [23]	**NP**: 10 **P1**: 10	**NP**: 64.4 (17.3) ^a^ **P1**: 55.4 (8.2)	**NP**: 30.8 (5.7) ^a^ **P1**: 26.6 (5.8)	**All**: no GI, hepatic, cardiac, renal disease, within 24 h; no APAP, anticholinergic drugs, drugs affecting GI function, no glaucoma, hypersensitive APAP, fasted before study **NP**: various gynecological procedures **P1**: termination of pregnancy	**NP**: - **P1**: -	Oral SP (tablet in 12.5 mL water)	20 mg/kg	*Blood:* Sampling on fixed times TOT: 140 Per patient: 7 (0–2 h)	2 h	GLC
**Galinski and Levy (1984)** [24]	**P3 = PP2**: 1	**P3 = PP2**: ~64	**P3 = PP2**: 27	-	**P3**: last day of pregnancy (9 months) **PP2**: 38 days	Oral tablet (glass of water)	0.975 g	*Urine:* Sampling on fixed times TOT: 19 Per patient: P3: 7 PP2: 12 P3: (0–14 h) PP2: (0–24 h)	P3: 14 h PP2: 24 h	HPLC
**Gin et al. (1991)** [25]	**PP1**: 8 **PP2**: 6/8	**PP1**: 65 (56-87) ^b^ **PP2**: -	**PP1**: 30 (27–37) ^b^ **PP2**: -	**All**: ASA score I, uncomplicated term pregnancies with normal vaginal delivery, no smoking, DM, pre-eclampsia, GI disease, opioids or other motility-influence drugs, no APAP 8 h before study. Fasted overnight for at least 6 h	**PP1**: day 1 (14–35 h) ^b^, day 3 (62–81 h) ^b^**PP2**: 6 weeks	Oral SP (soluble tablet in 100 mL water)	1.5 g	*Blood:* Sampling on fixed times TOT: 147 Per patient: 7 (0–2 h)	2 h	HPLC
**Kulo et al. (2012)** [31]	**D**: 8 **PP2**: 8/8	**D**: 78.5 (61–92.2) ^b^ **PP2**: 69 (52.2–88)	-	**D**: (semi)elective surgery	**PP3** = 10–15 weeks	IV	2 g LD, 1 g q6 h MD for 24 h	*Blood:* Sampling on fixed times TOT: 64 Per patient: 4 (0–6 h)	6 h	HPLC
**Kulo et al. (2012)** [30]	**D**: 28	**D**: 79 (57–110) ^b^	**D**: 31.5 (20–42) ^b^	(semi)elective surgery	Weeks of GA not reported	IV	2 g LD, 1 g q6 h MD for 24 h	*Blood:* Sampling on fixed times TOT: 115 Per patient: 4 (0–6 h)	6 h	HPLC
**Levy et al. (1994)** [29]	**NP***:* 20 **P1***:* 20	**NP***:* 61.1 (45–86) ^b^ **P1***:* 62.8 (47–85)	**NP***:* 30.0 (17–40) ^b^ **P1***:* 24.4 (17–40)	**All***:* fasted 4 h, no history of GI disease, no medication known to affect gastric emptying **NP***:* minor gynecological procedures **P1***:* suction termination	**NP**: - **P1***:* 8–12 weeks GA	Oral tablet	1.5 g (with 50 mL water)	*Blood:* Sampling on fixed times TOT: 360 Per patient: 9 (0–2 h)	2 h	HPLC
**Macfie et al. (1991)** [13]	**NP**: 15 **P1**: 15 **P2**: 15 **P3**: 15	**NP**: 63.13 (11.67) **P1**: 62.00 (9.97) **P2**: 60.20 (8.33) **P3**: 72.70 (7.20)	**NP**: 30 (22–42) ^b^ **P1**: 23 (16–35) **P2**: 21 (16–25) **P3**: 27 (22–35)	**P1**: suction termination pregnancy **P2**: prostaglandin extra amniotic termination **D**: before elective C-section	**P2**: 15–18 weeks GA **P3**: 37–40 weeks GA	Oral tablet	1.5 g (with 50 mL water)	*Blood:* sampling on fixed times TOT: 540 Per patient: 9 (0–2 h)	2 h	HPLC
**Miners et al. (1986)** [28]	**NP***:* 12 **P3**: 8	**NP**: 63 (7) **P3**: 68 (5)	**NP**: 19–31 ^c^ **P3**: 26–32	**All**: after overnight fast, permitted to eat 3 h after APAP dosing, no other medications 1 week before and after study, non-smokers, healthy	**NP**: *-* **P3**: 31–38 weeks GA	Oral tablet	1 g (with 150 mL water)	*Saliva:* Sampling on fixed times TOT: 200 Per patient: 10 (0–8 h) *Urine:* Sampling in fixed times TOT: 60 Per patient: 3 (0–24 h)	24 h	HPLC
**Nimmo et al. (1975)** [26]	**D**: 12 **PP2**: 10	-	**D** = **PP2**: 23 (17–34) ^b^	**All***:* no evidence of GI, hepatic, cardiovascular or renal disease. Fasted 4 h before and 2 h after APAP intake. **PP2**: no analgesic intake 16 h before APAP intake	**D**: 36 weeks GAP **P1**: 2–5 days	Oral tablet	1.5 g (with 200 mL water)	*Blood:* Sampling on fixed times TOT: 198 Per patient: 9 (0–8 h)	8 h	-
**Nitsche et al. (2016)** [27]	**D**: 34	**D**: 82 (62–100) ^b^	**D**: 32 (25–39) ^b^	No medical or obstetrical complications	**D**: 38–40 weeks GA	Oral	1 g	*Blood:* *-*	-	HPLC
**Rayburn et al. (1986)** [32]	**P3 = PP2**: 6	**P3**: 73.5 (66.7–72.6) ^b^ **PP2**: 62.1 (53.1–67.1)	**P3** = **PP2**: 29 (27–33) ^b^	Healthy (no hepatic or renal disease), non-smokers, no other drugs, fasted overnight	**P3**: 36 weeks of GA **PP2**: 6 weeks	Oral capsule	1 g (with 240 mL water)	*Blood:* Sampling on fixed times TOT:132 Per patient: 11 (0–12 h) *Urine:* Sampling on fixed times TOT: 60 Per patient: 5 (0–24 h)	24 h	HPLC
**Simpson et al. (1988)** [34]	**NP**: 14 **P1**: 16 **P2**: 12	**NP**: 59.6 (9.6) **P1**: 60.3 (11.5) **P2**: 57.8 (7.3)	**NP**: 31.8 (7.5) **P1**: 25.6 (8.0) **P2**: 24.8 (8.6)	**All**: healthy, no drugs. Fasted for 4 h before intake APAP. **NP**: minor gynecological surgery. **P1 = P2**: termination of pregnancy	**NP**: - **P1**: 8–11 weeks GA **P2**: 12–14 weeks GA	Oral tablet	1.5 g (with 150 mL water)	*Blood* Sampling on fixed times TOT: 196 Per patient: 7 (0–2 h)	2 h	Enzyme assay
**Stanley et al. (1995)** [33]	**P2 = P2/3 = P3 = PP2**: 10	-	**P**: 25 (18–31) ^b^	**All**: fasted overnight for 8 h. no smoking. Primigravida, PS. No chronic drug use, GI or renal disorders or risk factors for gestational diabetes	**P2**: 14–16 weeks GA **P2**/3: 26–28 weeks GA **P3**:36–38 weeks GA **PP3**: 8 weeks	Oral tablet	1.5 g (with 50 mL water)	*Blood* Sampling on fixed times TOT: 320 Per patient: 8 (0–2.5 h)	2.5 h	HPLC
**Whitehead et al. (1993)** [14]	**NP**: 32 **P1**: 18 **P2**: 10 **P3**: 36 **PP1 (day 1, 18–24 h) = PP1 (day 2, 24–48 h) = PP1 (day 5):** 17/36 **PP1 (2 h):** 12 **PP1 (day 2):** 12/12	**NP**: 58.3 (4.8) **P1**: 59.4 (6.0) **P2**: 62.0 (6.6) **P3**: 75.8 (14.4) **PP1**: 72.9 (9.3)	**NP**: 28.4 (4) **P1**: 24.2 (5.1) **P2**: 29.5 (2.6) **P3**: 26.2 (5.5) **PP1**: 27.6 (5.0	**All***:* healthy, no GI disease, no drugs influencing gastric motility. No obesity or multiple pregnancy. Fasting 4 h before APAP intake. **NP**: NOC **P1**: vaginal termination **P3**: vaginal delivery anticipated	**NP**: - **P1**: 8–10 weeks GA **P2**: 16–24 weeks GA **P3**: >34 weeks GA **PP1**: 2 h **PP1**: day 1 (18–24 h) **PP1**: day 2	Oral tablet	1.5 g (with 50 mL water)	*Blood* Sampling on fixed times TOT: 1503 Per patient:9 (0–2 h)	2 h	Enzyme assay
**Wong et al. (2002)** [35]	**P3**: 15 (11 for analysis)	**P3**: 74 (10)	**P3**: 31 (4)	No systemic disease (including gestation diabetes), multiple gestation, BMI > 30, use of medication known to affect gastric motility or secretion, no APAP in previous 48 h	**P3**: 37.2 (0.2) weeks GA	Oral SP	1.5 g (with 50 and 300 mL water, separated by 48 h)	*Blood:* sampling on fixed times TOT: 110 Per patient: 10 (0–2.5 h)	2.5 h	HPLC
**Wong et al. (2007)** [36]	**P3**: 10	**P3**: 123 (26)	**P3**: 27 (3)	No systemic disease (other than type II DM or gestational diabetes), no multiple gestation, no BMI < 35 kg/m^2^, use of medication known to affect gastric motility or secretion, no APAP in previous 48 h. Fasted overnight.	**P3**: 37.2 (0.6) weeks GA	Oral SP	1.5 g (with 50 and 300 mL water, separated by 48 h)	*Blood:* sampling on fixed times TOT: 90 Per patient: 9 (0–2 h)	2 h	HPLC

^a^ Arithmetic mean (SEM); ^b^ arithmetic mean (range); ^c^ range. **Abbreviations:** “-“ = not reported; APAP = acetaminophen; ASA = American Society of Anesthesiologists; BMI = body mass index (kg/m^2^); D = at delivery; DM = diabetes mellitus; GA = gestational age; GC = gas chromatography; GI = gastrointestinal; h = hours; HPLC = high-performance liquid chromatography; IV = intravenous; kg = kilogram; LD = loading dose; MD = maintenance dose; NOC = no oral contraceptives; NP = non-pregnant; P1 = first trimester; P2 = second trimester; P3 = third trimester; PG = pregnant; PP = postpartum; PP1 = acute postpartum (<2 weeks after delivery); PP2 = early postpartum (2–6 weeks after delivery); PP3 = late postpartum (>6 weeks after delivery); PS = singleton pregnancy, SEM = standard error of the mean; SP = suspension; TOT = all subjects.

**Table 2:** Summary of pregnancy-induced changes in the pharmacokinetics, obtained with NCA, of acetaminophen and its metabolites. Studies are presented in alphabetical order by author. Data are given in median [range], mean (SD) unless other use is specified.

Absorption Related Parameters	Beaulac-Baillargeon et al. (1993) [12]	Beaulac-Baillargeon et al. (1994) [22]	Clark and Seagel (1983) [23]	Galinski and Levy (1984) [24]	Gin et al. (1991) [25]	Levy et al. (1994) [29]
**C_max_ (µg/mL)**	NP: 12.91 P1: 7.07 P2: 4.83 P3: 5.28 *p* = -	NP: 11.6 (1.57) ^a^ P1: 11.16 (1.02) *p* > 0.05	NP: 19.6 (5.1) ^a^ P1: 15.5 (4.5) *p* = -		PP1 (day 1): 39.49 (8.44) ^a^ PP1 (day 3): 34.16 (3.64) PP2 (week 6): 37.34 (8.60) *p* > 0.05	**NP: 29.9 (11.5)** **P1: 23.3 (7.5)** ***p* < 0.05**
**t_max_ (min)**	NP: 30 P1: 30 P2: 35 P3: 30 *p* = -	NP: 46.0 (6.5) ^a^ P1: 48.0 (8.1) *p* > 0.05	NP: 60 P: 60 p = -		PP1 (day 1): 20.63 ^b^ PP1 (day 3): 20.63 PP2 (week 6): 27.50 *p* > 0.05	**NP: 48.0 (28.2)** **P1: 69 (29.0)** ***p* < 0.05**
**Gastric emptying t ½ (h)**		*-*				
**Distribution related parameters**						
**Vd (L)**	NP: 60.77 P1: 100.81 P2: 90.73 P3: 105.28 *p* = -	NP: 51 (3) ^a^ P1: 55 (5) *p* = -				
**Vd (L/kg)**	NP: 0.84 P1: 1.35 P2: 1.16 P3: 1.19 *p* = -	NP: 0.85 (0.05) ^a^ P1: 0.88 (0.08) *p* > 0.05				
**Elimination-related parameters**						
**t½ (h)**	NP: 1.84 P1: 1.58 P2: 1.59 P3: 1.42 *p* = -	**NP: 2.02 (0.08) ^a^** **P1: 1.62 (0.06)** ***p* < 0.005**				
**CL/F (L/h)**	NP: 22.79 P1: 44.05 P2: 39.54 P3: 51.38 *p* = -					
**CL/F (L/h/kg)**	NP: 0.32 P1: 0.59 P2: 0.50 P3: 0.58 *p* = -	**NP: 5.22 (0.46) ^a^** **P1: 7.14 (0.72)** ***p* = 0.03**				
**Urine excretion (mg (% of total APAP recovered))**		*-*		**P3**: APAP: 596 (100) PG: 387(65) PS: 196 (33) PU: 13 (2) **PP2:** APAP: 872 (100) PG: 449 (51) PS: 382 (44) PU: 41 (5)		

^a^ Arithmetic mean (SEM); ^b^ arithmetic mean calculated from results. Abbreviations: “-“ = not reported; APAP = acetaminophen; CL = clearance; C_max_ = maximum concentration; F = bioavailability; NP = non-pregnant; P1 = first trimester; P2 = second trimester; P3 = third trimester; PG = acetaminophen-glucuronide; PP1 = acute postpartum (<2 weeks after delivery); PP2 = early postpartum (2–6 weeks after delivery); PS = acetaminophen-sulphate; PU = unchanged acetaminophen; SEM = standard error of the mean; t _1/2_ = half-life; t_max_ = time at which the maximum concentration is achieved; Vd = volume of distribution.

**Table 3:** Summary of pregnancy-induced changes in the pharmacokinetics, obtained with NCA, of acetaminophen and its metabolites. Studies are presented in alphabetical order by author. Data are given in median [range], mean (SD) unless other use is specified.

Absorption-related Parameters	Macfie et al. (1991) [13]	Miners et al. (1986) [28]	Nimmo et al. (1975) [26]	Nitsche 2016 et al. [27]	Rayburn et al. [32]	Simpson et al. (1988) [34]
**C_max_ (µg/mL)**	NP: 28.19 (1.62) ^a^ P1: 23.01 (2.18) P2: 26.70 (2.54) P3: 28.10(2.12) *p* > 0.05		D: 21.0 (4.7) ^a^ PP1: 19.0 (1.9) *p* = -	D: 12.3	P3: 20.8 (6.9) PP2: 23.7 (6) *p* > 0.05	**NP: 34.4 (4.3) ^a^** P1: 26.8 (2.7) **P2: 21.4 (2.2)** ***p* < 0.05 P2/NP**
**t_max_ (min)**	NP: 61 (6.85) ^a^ P1: 74 (8.54) P2: 59 (9.32) P3: 45 (4.89) *p* > 0.05		D: 30PP1: 60 *p* = -		P3: 48 (24) PP2: 48 (24) *p* > 0.05	**NP: 45.0 (5.9) ^a^** **P1: 46.4 (8.1)** **P2: 71.9 (9.2)** ***p* < 0.05 P2/NP and P2/P1**
**Distribution-related parameters**						
**Vd (L)**		NP: 52.13 ^b^ P3: 59.44 *p* = -		D: 57.5	P3: 116. 93 ^b^ PP2: 68.89	
**Vd (L/kg)**		NP = 0.83 ^b^ P3 = 0.87 *p* = -		D: 0.70 ^b^	P3: 1.59 ^b^ PP2: 1.11 *p* = -	
**Elimination-related parameters**						
**t½ (h)**		**NP: 2.11 (0.27)** **P3: 1.52 (0.40)** ***p* < 0.002**		D: 1.4	P3: 3.7 (0.4) PP2: 3.1 (0.4) *p* > 0.05	
**CL/F (L/h)**		**APAP:** **NP: 17.12 (2.53)** **P3: 27.10 (5.73)** ***p*< 0.002** **PG:** **NP:9.95 (1.74)** **P3:17.37 (4.06)** ***p*< 0.002** **PS:** **NP**: 4.67 (0.68) P3: 5.57 (1.00) *p* > 0.05 **PU:** NP: 0.89 (0.27) P3: 1.18 (0.46) *p* > 0.05 **PO:** **NP: 1.60 (0.46) ^a^** **P3: 3.00 (0.53)** ***p* < 0.002**		D: 28.8	**P3: 21.9 (5.37)** **PP2: 15.4 (2.5)** ***p* < 0.05**	
**CL/F (L/h/kg)**		NP: 0.27 (0.04) ^b^ P3: 0.40 (0.08)		D: 0.35 ^b^	P3: 0.30 ^b^ PP2: 0.25	
**Urine excretion (%)**					**PG:** P3: 40.2 (21.5) PP2: 40.6 (20.7) *p* > 0.05 **PS:** P3: 17.8 (14.8) PP2: 24.7 (18.1) *p* > 0.05 **PU:** P3: 2.5 (2.1) PP2: 2.5 (2.3) *p* > 0.05 **PO:** P3: 9.0 ^c^ PP2: 5.9 *p* > 0.05	

^a^ Arithmetic mean (SEM); ^b^ calculated from results; ^c^ calculated from cystein and mercapturic adducts. Abbreviations: “-“ = not reported; APAP = acetaminophen; CL = clearance; C_max_ = maximum concentration; D = at delivery; F = bioavailability; NP = non-pregnant; P1 = first trimester; P2 = second trimester; P3 = third trimester; PG = acetaminophen-glucuronide; PO = oxidative metabolites of acetaminophen; PP2 = early postpartum (2–6 weeks after delivery); PS = acetaminophen-sulphate; PU = unchanged acetaminophen in urine; SEM = standard error of the mean; t _1/2_ = half-life; t_max_ = time at which the maximum concentration is achieved; Vd = volume of distribution.

**Table 4:** Summary of pregnancy-induced changes in the pharmacokinetic parameters, obtained with NCA, of acetaminophen and its metabolites. Studies are presented in alphabetical order by author. Data are given in median [range], mean (SD) unless other use is specified.

Absorption-Related Parameters	Stanley et al. (1995) [33]	Whitehead et al. (1993) [14]	Wong et al. (2007) [36]	Wong et al. (2002) [35]	Kulo et al. (2012) [31]	Kulo et al. (2012) [30]
**C_max_ (µg/mL)**	P2: 22 (5.1) P2/3: 20 (6.3) P3: 21 (5.0) PP3: 25 (5.2) *p* > 0.05	**NP: 20.8 (8.8–64.5) ^a^** P1: 21.3 (3.4–39.6) P2: 25.7 (16.5–33.1) P3: 21.0 (4.1–37.2) **PP1 (2 h): 10.1 (0.3–36.8)** ***p* < 0.01 PP1 (2 h)/NP** PP1 (day 1): 23.5(11.3–41.8) PP1 (day 2): 23.6(12.1–49.0) PP1 (day 5): 24.4(11.7–31.4) **PP1 (2 h) → (PP1, day 2): 36.6 (3.6–57.8)** ***p* < 0.01 PP1 (day 2)/PP1** **(2 h)**	P3 (50 mL): 9.9 (7.2) P3 (300 mL): 9.2 (3.6) *p* > 0.05	P3 (50 mL): 32.9 (11.2) P3 (300 mL): 30.7 (13.0) *p* > 0.05		D: 34.6 (12.7–46.6) ^a^
**t_max_ (min)**	P2: 114 (30) P2/3: 113 (22) P3: 118 (22) PP3: 102 (32) *p* > 0.05	**NP: 40 (10–120) ^a^** P1: 45 (10–120) P2: 30 (10–60) P3: 40 (10–120) **PP1 (2 h): 120** ***p* < 0.01 PP1 (2 h)/NP** PP1 (day 1): 30 (20–120) PP1 (day 2): 30 (10–90) PP1 (day 5): 35 (10–90) **PP1 (2 h) → (PP1, day 2): 40 (10–120)** ***p* < 0.01 PP1 (day 2)/PP1** **(2 h)**	P3 (50 mL): 53.4 (37.2) P3 (300 mL): 57.5 (38.8) *p* > 0.05	**P3 (50 mL): 40.9 (19.2)** **P3 (300 mL): 24.6 (12.1)** ***p* < 0.05**		
**Gastric emptying t ½ (h)**			P3: 50 mL: 0.53 (0.13) P3: 300 mL: 0.36 (0.1) *p* > 0.05	**P3 (50 mL): 0.55 (0.13)** **P3 (300 mL): 0.4 (0.1)** ***p* < 0.01**		
**Distribution-related parameters**						
**Vd (L)**					**D: 61.7 (43.5–75) ^a^** **PP3: 35.7 (29.5–59.3)** ***p* = 0.0234**	D: 58.3 (42.9–156) ^a^
**Vd (L/kg)**					D: 0.77 (0.7–0.87) ^a^ PP3: 0.59 (0.35–0.85) *p* > 0.05	D: 0.72 (0.52–15.6) ^a^
**Elimination-related parameters**						
**t½ (h)**					D: 1.9 (1.8–2.5) ^a^ PP3: 2.3 (1.4–3.6) *p* > 0.05	D: 1.93 (1.2–2.9) ^a^
**CL (L/h)**					**D: 22.19 (13.08-27.32) ^a^** **PP3: 11.31 (8.06–15.72)** ***p* = 0.0078**	D: 20.3 (11.8–62.8) ^a^
**CL (L/h/kg)**					**D: 0.29 (0.2-0.32) ^a^** **PP3: 0.17 (0.15–0.2)** ***p* = 0.0078**	D: 0.26 (0.15–0.79) ^b^
**Urine excretion (mg, % of total APAP recovered)**						

^a^ Arithmetic mean (range); ^b^ calculated from results. Abbreviations: “-“ = not reported; APAP = acetaminophen; CL = clearance; C_max_ = maximum concentration; D = at delivery; NP = non-pregnant; P1 = first trimester; P2 = second trimester; P3 = third trimester; PP = postpartum; PP1 = acute postpartum (<2 weeks after delivery); PP3 = late postpartum (>6 weeks after delivery); t _1/2_ = half-life; t_max_ = time at which the maximum concentration is achieved; Vd = volume of distribution.

**Table 5:** Details of the population PK models on acetaminophen in pregnancy. Data shown as mean (SD).

Model	Kulo et al. (2013) [15]	Allegaert et al. (2015) [16]
**Population and number of** **patients**	D: 39 PP: 8	D: 47 EP: 8/47 LP: 7/8 22 healthy female volunteers (with or without oral contraceptives)
**Patient Characteristics**		
**Age (years)**	D: 31.1 (5.28) PP: 32.13 (3.87)	D: 30.9 (5.3) EP: 32.1 (3.9) LP: 32.9 (4.1) Healthy women NOC: 31.1 (4.3) Healthy women OC: 23.5 (4.0)
**Body weight (kg)**	D: 79.37 (13.0) PP: 68.83 (11.7)	D: 79.7 (12.9) EP: 68.8 (11.2) LP: 32.9 (13.5) Healthy women NOC: 63.9 (6.6) Healthy women OC: 58.8 (8.9)
**Gestational age (weeks)**	D: 35.9 (4.09)	D: 45% < 37 weeks
**Metabolites**	APAP-U (blood, urine) APAP-G (urine) APAP-S (urine) APAP-O (estimated)	APAP-U (blood, urine) APAP-G (urine) APAP-S (urine) APAP-O (not estimated, not measured; ignored)
**Model**	Non-linear mixed-effect modelling APAP-G and APAP-S Vd = 18% of Vc APAP	Non-linear mixed-effect modelling APAP-G and APAP-S Vd = 18% of Vc APAP
**Covariate modelling**	Comprehensive covariate analysis Covariates vs. individual post hoc parameter estimates Covariates vs. weighted residuals	Comprehensive covariate analysis Covariates vs. individual post hoc parameter estimates Covariates vs. weighted residuals
**Internal model evaluation and validation**	Observed vs. individual predictions Observed vs. population predictions Conditional weighted residuals vs. time Conditional weighted residuals vs. population predictions CI of parameter estimates Correlation matrix Visual improvement of the individual plots Bootstrap (*n* = 250)	Observed vs. individual predictions Observed vs. population predictions Conditional weighted residuals vs. time Conditional weighted residuals vs. population predictions CL of parameter estimates Correlation matrixVisual improvement of the individual plots Bootstrap (*n* = 250)
**External model evaluation and validation**	None	None

Abbreviations: APAP = acetaminophen; APAP-G = acetaminophen-glucuronide; APAP-O = oxidative metabolites; APAP-S = acetaminophen-sulphate; APAP-U = unchanged acetaminophen; CL = clearance; D = at delivery; EP = early postpartum (10–15 weeks after delivery); LP = late postpartum (1 year after delivery); NOC = no oral contraceptives; OC = oral contraceptives; PP = postpartum (12 weeks after delivery); Vc = central compartment volume of distribution; Vd = volume of distribution.

## Data Availability

Not applicable.
